# Mammary Ductal Carcinoma In Situ: A Fresh Look at Architectural Patterns

**DOI:** 10.1155/2012/979521

**Published:** 2012-02-29

**Authors:** Gabriel Scripcaru, Ibrahim M. Zardawi

**Affiliations:** ^1^Department of Pathology, Royal Darwin Hospital, Tiwi, NT 2011, Australia; ^2^Pathology North, Taree, NSW 2430, Australia; ^3^Academic Pathology, The University of Newcastle, NSW, Australia

## Abstract

Mammary ductal carcinoma in-situ (DCIS), a malignant appearing lesion on cytological and histological grounds, is in fact a non-obligate precancer. DCIS is difficult to manage and is sometimes treated more aggressively than invasive carcinoma. Although most DCIS classifications take into account the architectural growth pattern, when it comes to architecture, the literature is full of contradictory information. We examined 289 breast cancers and found DCIS in 265 of the cases. The majority of the DCIS cases were seen in the setting of invasive cancer and only 9% of the cases represented pure DCIS with no invasive cancer. The DCIS commonly displayed a mixed pattern with micropapillary, cribriform and solid components with the micropapillary type being the rarest, occurring seldom on its own. A continuum of growth with a micropapillary pattern evolving into a cribriform type could be seen in some of the cases. This may explain some of the conflicting information, in the literature, regarding the different architectural types of DCIS. The comedo-pattern of necrosis could be seen in all types of DCIS. We therefore conclude that the study of the determinants of growth pattern in DCIS would be the key to unravelling the diverse, often non-concordant evidence one encounters in the literature.

## 1. Introduction

Classifying and managing DCIS has always been a thorny issue, often dividing various groups of pathologists around the world [1]. 

Amongst DCIS features, the architectural pattern, its prognostic value, and role in grading DCIS have also been stirring sufficient controversy. The current literature on the subject accepts the existence of 3 major architectural patterns of DCIS, namely, the solid, cribriform, and micropapillary patterns [2]. 

The clinging or flat type is not universally accepted as fully developed DCIS. It has been variably considered as an early micropapillary DCIS or even a subvariant of the atypical ductal hyperplasia [3]. 

Other special types of DCIS, such as the apocrine, the endocrine (argyrophilic), and signet ring DCIS, are all defined on histological criteria, rather than architectural pattern and they actually belong to the solid pattern of growth.

With respect to grading, it is universally accepted that the nuclear grade is the essential feature, recurring in all classification systems previously proposed and currently in use [4]. 

An association, albeit inconsistent, exists between the nuclear grade and the architectural growth pattern. It is generally accepted that most micropapillary and cribriform in situ carcinomas are of low nuclear grade and relatively indolent [5]. However, in a recent publication by Fisher and colleagues, micropapillary DCIS was found to be associated with both ipsilateral and contralateral recurrence of malignancy in a statistically significant number of cases [6]. 

As this result appears somewhat puzzling in the light of our present knowledge and understanding of DCIS, we decided to have a new, fresh look at all cases of DCIS reported during the past approximately 10 years at the Department of Pathology of the Royal Darwin Hospital, NT, Australia and report our findings.

## 2. Materials and Methods

All cases of DCIS reported at the Royal Darwin Hospital, Northern Territory, Australia between January 2001 and September 2010, representing 60% of all breast cancers in the NT were retrospectively reviewed. In order to capture all the cases, including those that may have been incorrectly coded, we verified all breast tissue reports and then selected for active review all cases of DCIS. The architectural and cytological aspects of DCIS were assessed. The presence or absence of necrosis was also evaluated.

### 2.1. Architecture

The 3 main types of DCIS, according to the architectural growth pattern (micropapillary, cribriform, and solid) were assessed. Architecturally DCIS was divided into single, when >90% of the in situ tumour displayed one architectural pattern, and mixed when the dominant pattern constituted <90% of the in situ carcinoma.

### 2.2. Nuclear Grade

Nuclear grading is based on the size of malignant cells nuclei in comparison to normal ductal epithelial cells. Grade 1 is applied when the nuclei of the malignant cell are between 1.5 and 2 times that of normal ductal epithelial cell. Grade 2 is applied when the nuclei of the malignant cell are between 2 and 2.5 times that of normal ductal epithelial cell. Grade 3 is applied when the nuclei of the malignant cell are greater than 2.5 times that of normal ductal epithelial cell [7].

### 2.3. Necrosis

Any necrosis in DCIS was recorded. Minimal necrosis was labelled as necrosis, not otherwise specified. The term comedonecrosis, which is poorly defined in the literature, was applied when significant necrosis, creating an appearance similar the comedos, seen in cutaneous acne, was noted in ducts with DCIS.

## 3. Results

A total of 289 breast carcinomas had been received at the Royal Darwin Hospital during the period of the study. These consisted of 265 invasive and 24 pure in situ cancers.

These cancers consisted of 231 infiltrating duct carcinomas of no special type, 24 infiltrating lobular carcinomas and 10 carcinomas of special type. Of these special breast cancers, 5 were pure mucinous carcinomas, 2 were invasive papillary carcinomas, 2 were tubular carcinomas, and 1 was medullary carcinoma.

DCIS was present in 133/265 (50.18%) of the invasive ductal carcinomas. The proportion of invasive ductal carcinomas containing DCIS varied from year to year, ranging from 16/26 (62%) in 2002 to 8/26 (31%) in 2007. The reason for this variation is not clear.

### 3.1. DCIS Subtypes

Table 1 also shows the frequency of pure DCIS and DCIS associated with invasive cancer.

Of the 157 cases of DCIS (pure DCIS and invasive cancers with DCIS), 91 (58%) displayed a single growth pattern and 66 (42%) showed a mixed growth pattern (Table 1).

Of the 91 cases of DCIS with a single growth pattern, 4 (5%) were micropapillary, 23 (25%) were cribriform, 61 (67%) were solid, and 3 (3%) were encysted papillary (Table 1).

Of the 66 mixed type DCIS cases, all 3 growth patterns (micropapillary, cribriform, and solid) were noted in 11 (17%) of the cases. Micropapillary and cribriform was present in 14 (21%), micropapillary and solid was seen in 11 (17%), and cribriform and solid was identified in 30 (45%) of the cases (Table 1).

Most of the pure in situ carcinomas were of the mixed type and there were no cases of single pattern micropapillary DCIS (Table 2).

Overall, a micropapillary pattern was seen in 40/157 (25%), a cribriform component in 78/157 (50%), a solid component in 113/157 (72%), and macropapillary (encysted papillary carcinoma) in 3 (1.9%) of the DCIS cases (Table 2).

### 3.2. Comedonecrosis

Comedonecrosis was present in 18/40 (45%) of the micropapillary, 67/113 (59%) of the solid, and 20/78 (26%) of the cribriform cases of DCIS. These findings show that comedonecrosis is more likely to be seen in micropapillary and solid types DCIS than in the cribriform type. This association is statistically significant with *P* values of 0.033 and 0.00004, respectively. At the same time, no statistically significant difference in comedonecrosis occurrence was noted between the micropapillary and solid DCIS (*P* = 0.117).

### 3.3. Nuclear Grade

Of the 157 cases of DCIS high nuclear grade was recognised in 70 (45%), intermediate nuclear grade in 65 (41%), and low nuclear grade in 22 (14%) of the cases (Table 3).

### 3.4. Nuclear Grade versus Architectural Type of DCIS

Of the 40 cases of micropapillary DCIS, 2 (5%) were low grade, 18 (45%) were intermediate grade, and 20 (50%) were high grade (Table 3).

Of the 78 cases of cribriform DCIS, 17 (22%) were low grade, 39 (50%) were intermediate grade, and 22 (28%) were high grade (Table 3).

Of the 113 cases of solid DCIS, 8 (7%) were low grade, 47 (42%) were intermediate grade, and 58 (51%) were high grade (Table 3).

These results show a statistically significant difference between the presence of high nuclear grade (grade 3) in the solid and micropapillary types of DCIS compared to the cribriform type (*P* = 0.0008, and *P* = 0.019 resp.). On the other hand, no statistically significant nuclear grade differences were noted between the micropapillary and solid types (*P* = 0.884).

All three cases of macropapillary (encysted papillary carcinoma) were low grade (Table 3).

### 3.5. Micropapillary DCIS

Overall, we identified 40 cases with a micropapillary DCIS component (Table 2). Thirty-six of these were mixed with other growth patterns and 4 had only micropapillary growth pattern. In the cases of DCIS with no invasive cancer, there was a micropapillary component in 9 cases; all mixed with other growth patterns. The remaining 31 cases of micropapillary DCIS had an associated invasive component. Of these, 4 had only a micropapillary growth and in 27 the micropapillary pattern was mixed with other growth patterns.

Of all the 91 cases of DCIS with a single growth pattern, 4 (4%) had only micropapillary growth (Table 1). Of the 24 cases of pure DCIS, none was only micropapillary but 9 had a micropapillary component, mixed with other architectural patterns. Of these, 6 cases were high grade, 2 cases were intermediate grade, and 1 case was low grade (Table 3).

The age of patients who had a micropapillary component ranged from 38 years to 88 years with a median of 50 years. The age range for the low grade was 52 years to 63 years, for the intermediate grade was 38 years to 88 years, and for the high grade was 40 years to 80 years.

The invasive component of cases that included a micropapillary type DCIS was grade 3 in 22.6% (7/31), grade 2 in 38.7% (12/31), and grade 1 in 38.7% (12/31) of the cases.

Of the 4 cases with micropapillary DCIS not associated with any other type of DCIS (pure micropapillary type), 3 were intermediate grade and 1 was low grade. There were no high grade cases (Table 3).

The micropapillary DCIS represented only a minor component (<25%) in 9/36 of the cases in which it appeared in combination with the other types of DCIS.

### 3.6. Concordance between the Original Assessment and the Current Review

The cases had been reported by 6 pathologists, of which three were responsible for reporting 94% of cases. The overall concordance for all cases of DCIS, in respect to nuclear grade, regardless of architectural pattern, was very good, with a Kappa score of 0.87.

In cases with micropapillary component there was a good concordance between our evaluation and the initial report with a Kappa score of 0.61. The nonconcordant cases, which differed by 1 grade, were all between low to intermediate grade; none involved a high grade.

The concordance rate for cases not including a micropapillary pattern was also very good with a Kappa score of 0.88.

## 4. Discussion

It is beyond the scope of this study to chronologically recapitulate all aspects of this complex histopathological entity.

Regarding the micropapillary DCIS, our results are surprising, but may explain partly the increased correlation with breast carcinoma recurrence identified by Fisher et al. [6].

Fisher and colleagues reviewing DCIS from 1456 patients enrolled in the National Surgical Adjuvant Breast and Bowel Project (NSABP) protocol B-24 to determine predictors for ipsilateral breast tumour recurrences and contralateral breast cancers, after a median follow-up time of 10.5 years, found ductal comedonecrosis, micropapillary histological tumour type to be independent high risk factors for ipsilateral breast tumour recurrence and for contralateral breast cancers [6]. 

In a recent article, Castellano et al. have shown the nuclear grade to be crucial in determining the biology of micropapillary DCIS. They also showed that high nuclear grade micropapillary DCIS more frequently overexpressed HER2, showed a higher proliferation index, and displayed necrosis and microinvasion. Logistic regression analysis confirmed high nuclear grade (odds ratio, 6.86; confidence interval, 1.40–33.57) as the only parameter associated with elevated risk of local recurrence after breast-conserving surgery. However, the recurrence rate of 19 micropapillary DCIS, which were part of a cohort of 338 consecutive DCIS, was significantly higher (log-rank test, *P* = 0.019) than that of nonmicropapillary, independent of nuclear grade. The authors concluded that although nuclear grade may significantly influence the biological behaviour of micropapillary ductal carcinoma in situ, micropapillary growth pattern represents a risk factor for local recurrence after breast-conserving surgery [8]. 

The micropapillary pattern of DCIS is by far the rarest one in our study. It was seldom seen on its own. While the number of cases in which the micropapillary growth pattern constitutes the only pattern was very small in our study, it may not be a coincidence that none of these cases was high nuclear grade and none was associated with necrosis. This finding would be consistent with the dogma defining micropapillary DCIS as a predominantly low grade DCIS.

The frequent association between micropapillary DCIS and necrosis may explain why authors, considering comedonecrosis a separate pattern of DCIS, have been discarding all those cases also displaying comedonecrosis from the micropapillary group [8]. 

Another notion on which all publications seem to agree is the fact that the solid variant tends to be associated with high grade DCIS, whereas the micropapillary and cribriform variants most often form a low grade DCIS [5]. 

We believe comedocarcinoma is not a separate type of DCIS and should not be used as such. In other words, each DCIS should be given an architectural pattern label and the presence or absence of necrosis should be described separately.

In all the cases of DCIS with a pure micropapillary growth pattern, the nuclear grade was low or intermediate. This may mean that those cases of low grade DCIS that may have a certain genetic makeup will maintain an indolent course, whereas the more aggressive ones will undergo the changes described above and assume a mixed growth pattern with comedonecrosis.

Because of this supposed evolution, the micropapillary component may represent a minor proportion of the entire DCIS at the time the tissue is removed for histological evaluation.

If comedocarcinoma is considered a separate type of DCIS in which the architectural pattern is neglected and not reported, then our results, with only 4 cases of micropapillary DCIS, are all in keeping with the old dogma that micropapillary DCIS is a low or intermediate grade with no necrosis.

We agree with Fisher and colleagues [6] in considering comedocarcinoma a separate feature, rather than a histological type.

Pinder and O'Malley [9] hit the nail on the head and explain that comedonecrosis may be seen in association with any DCIS architectural type, and that it is not a type itself, as it is “neither a grade nor an architectur.” So they clearly recommend that the term comedo-type DCIS should not be used as a characterisation of growth pattern. This is exactly what we feel and what our study supports.

Also in keeping with our view is the fact that the rare, special type DCIS, namely, the signet ring, endocrine, and other types, also have a growth pattern that is, the solid one, even if the cells show “specific” cytological features.

Our results show that over 40% (66/157) of the DCIS cases are not of pure architectural type, but in fact they are mixed. Approximately half of the solid (51%) and micropapillary (50%) cases in our series are high grade. These values are statistically significant when compared to the occurrence of high grade cribriform DCIS, of which only 28% are high grade.

Just under half (45%) of the micropapillary and nearly two-thirds (67%) of the solid cases of DCIS are associated with comedonecrosis, while only a quarter (26%) of the cribriform cases of DCIS are associated with comedonecrosis. These differences are also statistically significant as shown above. The micropapillary type is the rarest one, occurring seldom as a pure form with less than 3% of all cases of DCIS and less than 5% of DCIS cases with a single pattern being pure micropapillary ones.

Slightly more than half (54%) of the solid and almost a third (30%) of the cribriform cases occur on their own, whereas in only 10% of the cases the micropapillary type occurs on its own.

Low grade micropapillary and solid DCIS were very rare in our series (5% and 7%, resp.).

Our results can be translated into the newly accepted DIN system by replacing the nuclear grade value with the equivalent DIN [10].

## 5. Conclusions

After carefully observing and analysing our data, we draw the conclusion that in many cases the pattern of growth may be a continuum, starting as micropapillary, with papillae then either joining one another to form arches resembling the cribriform pattern or even continuing to proliferate until a solid sheet of cells fills the entire lumen.

As necrosis occurs toward the tips or on the sides of the micropapillary structures, the lesion becomes intermediate grade. With progression, nuclear pleomorphism and a comedo-type necrosis appear, and the lesion then qualifies as a high grade DCIS.

We therefore believe the study of the determinants of growth pattern in DCIS would be the key to unravelling the diverse, often nonconcordant evidence one encounters in the literature.

## Figures and Tables

**Table 1:** DCIS pure and with invasive cancer and growth pattern.

DCIS growth pattern (157 cases)	
Single	91 (58%)
Mixed	66 (42%)
DCIS with a single growth pattern (91 cases)	
Micropapillary	4 (5%)
Cribriform	23 (25%)
Solid	61 (67%)
Macropapillary/encysted papillary	3 (3%)
DCIS with a mixed growth pattern (66 cases)	
Micropapillary, cribriform, and solid	11 (17%)
Micropapillary and cribriform	14 (21%)
Micropapillary and solid	11 (17%)
Cribriform and solid	30 (45%)

**Table 2:** DCIS by growth pattern.

Micropapillary (40 cases)	
Pure micropapillary DCIS without cancer	0
Pure micropapillary DCIS with invasive cancer	4
Cribriform (78 cases)	
Pure cribriform DCIS without cancer	2
Pure cribriform DCIS with invasive cancer	21
Solid (113 cases)	
Pure solid DCIS without cancer	7
Pure solid DCIS with invasive cancer	54
Mixed DCIS (66 cases)	
Mixed DCIS without cancer	12
Mixed DCIS with invasive cancer	54
Macropapillary (3 cases)	
Pure macropapillary DCIS without cancer	3
Pure macropapillary DCIS with invasive cancer	0

**Table 3:** DCIS by nuclear grade.

All cases with any micropapillary DCIS component (40 cases)	
Low grade	2 (5%)
Intermediate grade	18 (45%)
High grade	20 (50%)
All cases with any cribriform DCIS component (78 cases)	
Low grade	17 (22%)
Intermediate grade	39 (50%)
High grade	22 (28%)
All cases with any solid DCIS component (113 cases)	
Low grade	8 (7%)
Intermediate grade	47 (42%)
High grade	58 (51%)
All cases with any mixed DCIS component (66cases)	
Low grade	8 (12%)
Intermediate grade	30 (45%)
High grade	28 (43%)
Macropapillary (3 cases)	
Low grade	3 (100%)
Intermediate grade	0 (0%)
High grade	0 (0%)
